# Prevalence and risk estimates of *Cryptosporidium* oocysts infection associated with consumption of raw-eaten vegetables in Maiduguri metropolis LGAs, Northeast Nigeria

**DOI:** 10.1038/s41598-023-49451-0

**Published:** 2023-12-27

**Authors:** A. S. Saidu, S. Mohammed, S. G. Adamu, M. A. Sadiq, A. O. Tijjani, H. I. Musa, S. M. Jajere, M. D. Goni, A. Muhammed, M. D. Idriss

**Affiliations:** 1 Department of Veterinary Public Health and Preventive Medicine, Faculty of Veterinary Medicine, University of Maiduguri, Maiduguri, 600001 Borno State Nigeria; 2 Public Health and Zoonoses Research Group, Faculty of Veterinary Medicine, Universiti Malaysia Kelantan, 16100 Kelantan, Pengkalan Chepa Malaysia; 3 Department of Veterinary Laboratory Diagnostics, Faculty of Veterinary Medicine, Universiti Putra Malaysia (UPM), 43400 Serdang, Selangor Malaysia; 4 Institute for Artificial Intelligence and Big Data, Universiti Malaysia Kelantan, 16100 Kota Bharu, Kelantan Malaysia

**Keywords:** Diseases, Health occupations, Risk factors

## Abstract

*Cryptosporidium* is one of the most important protozoan parasitic pathogens, and it is a common cause of diarrhoea in humans, domestic animals, and wild vertebrates and has serious public health threats. A cross-sectional study was designed to determine the prevalence of *Cryptosporidium* oocysts in raw-eaten vegetables in Maiduguri Metropolitan Council (MMC) and Jere Local Government Areas (LGAs). A total of 400 samples were collected from four (4) different locations, namely Tashan Bama, Gomboru*,* and Monday Market*s* (n = 100), while fifty (n = 50) each from 202-Vegetable-Vendors and Unimaid Commercials. A total of 16 visits were conducted in all the sampling areas (twenty-five samples per visit). The *Cryptosporidium* oocysts were detected using the Modified Ziehl–Neelsen Staining Technique. The locations, sources, and types of raw-eaten vegetables were also assessed. The oocysts were confirmed (100×) as bright pink spherules. Data generated were analyzed using IBM-SPSS V23.0, and p ≤ 0.05 was considered significant. Out of the total samples (n = 400) analyzed, cabbage appeared to have the highest number of 10 (12.5%) of *Cryptosporidium* oocysts detected, while Tomato and garden egg had 9 (11.3%) and 1 (1.2%), respectively. There was a statistically significant association (χ^2^ = 12.5, P = 0.014) between the presence of *Cryptosporidium* oocysts in raw-eaten vegetables and vegetable types. Among the sources of the vegetables sampled, Alau had the highest number of *Cryptosporidium* oocysts, 15 (12.5%), followed by Kilari-Abdullahi and Zabarmari sources with 4 (10.0%) and 4 (5.0%), respectively. However, Jetete appeared to have the least number 2 (2.5%) of oocysts, and there was a statistically significant association (χ^2^= 10.4, P = 0.034) between the presence of *Cryptosporidium* oocysts and the sources of vegetables and fruits. The study concludes that the raw-eaten vegetables sampled from Maiduguri Metropolis were contaminated with *Cryptosporidium* oocysts. The study recommends that all raw-eaten vegetables should be from cleaned sources and washed before consumption. Consumers should be enlightened on the hygienic measures in the food chain in line with the Hazard Analysis and Critical Control Points (HACCP) principles.

## Introduction

*Cryptosporidium* is a protozoan parasite that belongs to the Phylum Apicomplexa and Family *Cryptosporidiidae*. It is also a common cause of diarrhoea in domestic animals, wild vertebrates and humans^1^. This is particularly amongst immunocompromised patients such as HIV/AIDS^2,3^, young and elderly individuals undergoing cancer chemotherapy, and other conditions that suppress immunity, such as malnutrition^4^.

*Cryptosporidium* is known to be responsible for 8–19% of cases of diarrheal disease in low-medium income countries, causing a wide range of infections in various vertebrate hosts^5^. *Cryptosporidium* attacks the intestinal cells, causing self-limiting diarrhoea and respiratory diseases in the vertebrate hosts^6^. *Cryptosporidium hominis* or *C. parvum* were incriminated as the cause of cryptosporidiosis in humans and several other mammals, which play a significant role in zoonotic transmission^7,8^. Various cases of human cryptosporidiosis have been reported in Kaduna, north-west^9,10^, north-central^11,12^, South East^13^ and Southwestern^14,15^ parts of Nigeria.

The disease is a major public health threat in developing and developed countries^16^, where raw vegetables are common vehicles for transmitting the parasite. Fresh vegetables are an important part of a healthy diet, and such leafy plants are eaten raw or lightly cooked to preserve taste in many countries. However, this practice favours the likelihood of contracting many foodborne diseases, especially *Cryptosporidium* infections^17^. This is because most of the water used for irrigation farming of raw-eaten vegetables in developing countries such as Nigeria is from contaminated sources such as abattoir effluents and liquid waste, rich sources of parasitic, viral, and bacterial load, in addition to habits of indiscriminate defecation as well as the absence or obsolete effluent pumping room in most abattoir settings in Nigeria, which is another confounder to the high burden of *Cryptosporidium* infections in the country^8^. The sources of zoonotic contamination of vegetables with *Cryptosporidium* oocysts are usually faeces, fecal-contaminated soil, or contaminated water^18^. In recent years, there has been an increase in cases of foodborne illness linked to the consumption of fresh vegetables. The most evidence was from the study conducted by^9^, who reported the presence of *Cryptosporidium* oocysts in raw-eaten vegetables in Northwestern Nigeria. Earlier studies in different parts of the globe have revealed that consumption of contaminated raw vegetables and fruits was attributed to the presence of gastrointestinal parasites such as *Cryptosporidium*, *Entamoeba*, *Giardia*, tapeworms, and roundworms in humans^19–21^. In many developing countries, poor hygiene and low living conditions are responsible for the increased risk of foodborne parasitic infections, especially in raw-eaten vegetables^20^. In Maiduguri, previous studies have shown that raw vegetables sold for human consumption were heavily contaminated with helminth eggs and larvae, and the prevalence of helminth eggs was associated with vegetable types^22^. However, to our knowledge, there is no published document on the contamination of raw-eaten vegetables with *Cryptosporidium* oocysts in Maiduguri, Borno state, Northeastern Nigeria. This study will provide baseline data on the level of vegetable contamination with *Cryptosporidium* oocysts and highlight the public health hazards associated with the consumption of improperly sanitized raw-eaten vegetables. This study aimed to determine the prevalence of *Cryptosporidium* species in raw-eaten vegetables in selected LGAs in Maiduguri Metropolis.

## Materials and methods

### Study area

The study was conducted in Maiduguri Metropolitan Council (MMC) and Jere LGAs of Borno state. Maiduguri is the capital city of Borno state in northeastern Nigeria. The study area was situated along the seasonal *Ngadda* River, which disappears into the *Firkin’s* swamp in the study around Lake Chad. The average temperature was 39 °C. Maiduguri is estimated to have a population of 1,907,600 as of ^23^. The state has three international borders Chad Republic, Niger, and Cameroon favouring its socio-economic activities and international trade. The climate is semi-arid with about two climatic seasons: the wet season, which starts from April to October with an average daily temperature of about 38.8 °C and the dry season, which starts from November to March with an average daily temperature of about 36.8 °C.

### Study design

**Figure 1:**
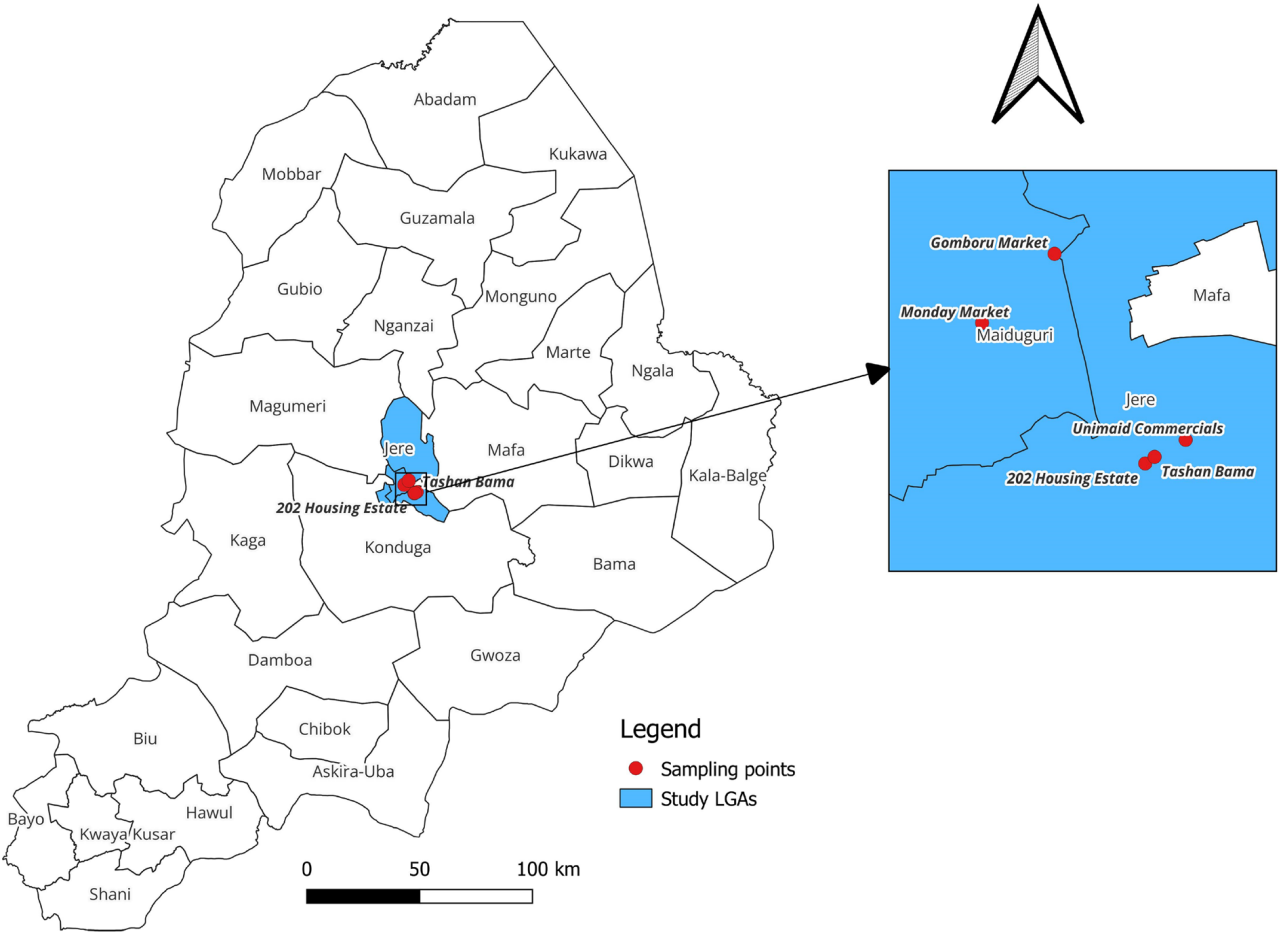
The map of Borno State showing the study areas and the sampling points in MMC and Jere LGAs sources: QGIS V3.28.1: Personal\C:\Users\Dr. Adamu-REDISSE\OneDrive\Desktop\Figure2_Study Maps.jpg.

A cross-sectional study was designed to establish the prevalence of *Cryptosporidium* oocysts raw-eaten vegetables in MMC and Jere LGAs. The selection was based on the availability of raw-eaten vegetables in those locations. A total of 400 samples were collected from five (5) different locations, two of which were from MMC and three from Jere LGA. The Monday Market and Gomboru Vegetable Vendors are from MMC, whereas Tashan Bama, 202 Vegetable Vendors and Unimaid Commercials are from Jere LGA (Fig. 1).

Maiduguri is located on latitude 11.8311" northland and Longitude 13.1510° east of the equator. It is the largest city in the northeastern part of Nigeria and has a landmass of 132 km^2^. The city is situated at about 325 m above sea level. According to the National Population Commission, the population of the city was estimated at 731,700 in 2015, with a projected population density of 5543.2 inhabitants. No human sample was involved in this study.

### Sample size determination

A total of 384 samples were determined based on the 50% proportion described by^24^ due to the absence of previous prevalence of *Cryptosporidium* oocysts in the study area, as presented below.

Using the formula for sample size estimation as described by^24^:

$${\text{n}} = {\text{ Z}}^{{2}} {\text{PQ}}/{\text{e}}^{{2}}$$

where; n is the sample size; Z is the corresponding value of confidence level (1.96 for 95% CI); P is the estimated value for the proportion of sample 50%; Q = 1 − P

$${\text{n}} = \, \left( {{1}.{96}} \right)^{{2}} \times \, 0.{5}\left( {{1} - 0.{5}} \right)/\left( {0.0{5}} \right)^{{2}}$$

$$n=\frac{\left(1.96\right)2 \times 0.5 (1-0.5) }{\left(0.05\right) 2}\times 100$$

$${\text{n}} = {3}.{842} \times 0.{25}/0.00{25 } = \, 0.{96}0{5}/0.00{25},$$

$${\text{n}} = { 384}$$

A total of 384 raw vegetables are required, but for standard error estimation and precision, 400 samples were collected in this study.

### Sample collection and transportation

Samples were randomly collected from Tashan Bama, 202 Vegetable Vendors, University Commercial, Gomboru Market and Monday Market in Maiduguri, Borno state. A proportionate sampling was applied in which one hundred vegetable samples were collected from Tashan Bama (n = 100), Gomboru Market (n = 100), and Monday Market (n = 100), and fifty raw vegetable samples each from 202-Vegetable Vendors (n = 50) and University commercials (n = 50). Five (5) available vegetables and fruits sampled for this study are Tomatoes, Carrots, Cabbage, Garden eggs, and Cucumber. A total of 16 visits were conducted in all the sampling areas (twenty-five samples per visit) until the determined sample size of 400 was reached. Samples were collected during the morning, placed into clean polythene bags, labelled, and immediately transported to the Parasitology Laboratory in the Department of Veterinary Parasitology and Entomology, University of Maiduguri, for further processing. The raw-vegetable samples were then processed to detect *Cryptosporidium* oocysts using floatation, sedimentation and *Ziehl–Neelsen* Techniques and further microscopy.

### Laboratory analysis

#### Washing of raw vegetables with normal saline

Each collected sample was placed into a plastic container and washed in a 250 ml beaker containing 150 ml of physiological saline solution (0.95% NaCl), as described by Kemajou et al.^25^.

#### Sedimentation and floatation techniques

After washing the raw vegetables with normal saline, it was left in a rubber cup for 24 h to sediment. The supernatant was discarded, and the sediment was mixed with a saturated salt solution before putting it into the sample bottle. Each sample bottle was then topped to the brim with the flotation medium to form a meniscus. A glass slide was placed on the sample bottle for 5 min, which was removed and allowed to air-dry as described by Kemajou et al.^25^.

#### Preparation of acid alcohol and modified Ziehl–Neelsen stain

Acid alcohol (1%) was prepared using 20.20 mL of the absolute alcohol of 99% concentration mixed with 1979.80 mL of distilled water. All methods were carried out following Sekar et al. guidelines^26^.

#### Modified Ziehl–Neelsen staining technique

Air-dried slides were stained using a modified *Ziehl–Neelsen* technique^27^ by fixing the air-dried slides in methanol for 2–3 min. The slides were flooded with cold carbol fuchsin for 5–10 min and then with 1% hydrochloric-acid ethanol until colour ceased to flow out and rinsed in tap water. It was then counter-stained with 0.25% methylene blue for 30 s, then rinsed in tap water and air-dried. Each slide was examined using a light microscope at  40× and  100× magnifications. The *Cryptosporidium* oocysts appeared as bright pink spherules. Oocysts were confirmed under a higher-powered magnification (100×).

### Data analysis

Data was presented in the form of charts and tables. All variables location, type, and source of raw-eaten vegetables) generated were analyzed using IBM-SPSS V23.0 to establish a possible association with the presence of *Cryptosporidium* oocysts using a chi-squared test of association. A value of P ≤ 0.05 was considered significant throughout the study.

### Ethics approval and consent to participate

Verbal consent was sought and approval was given to us by the leadership of the vegetable vendors before the commencement of the study. No human sample was involved in this study.

## Results

### Prevalence of *Cryptosporidium *oocysts in different raw eaten vegetables in MMC and Jere LGAs

**Figure 2:**
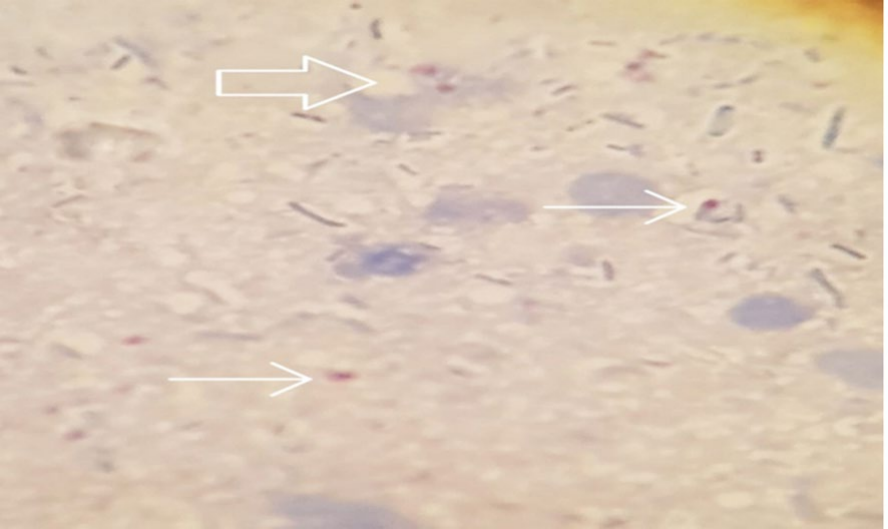
Typical Cryptosporidium oocysts detected in raw eaten vegetables sampled from Maiduguri Metropolis (MMC and Jere) using modified *Ziehl–Neelsen* staining technique (magnification-×100).

Out of the 400 raw-eaten vegetables and fruits collected, 28.0 (7.0%) were detected to be contaminated with *Cryptosporidium* oocysts using a modified *Ziehl–Nelsen* staining technique. The typical *Cryptosporidium* oocysts detected in raw-eaten vegetables in this study are presented in Fig. 2.

The overall prevalence of *cryptosporidium* oocysts in raw-eaten vegetables found in this study was 7.0%. Among all the vegetables sampled, Cabbage appeared to have the highest number 10.0 (12.5%) of *Cryptosporidium* oocysts detected, followed by Tomato with 9.0 (11.3%), and the lowest number was recorded in garden egg 1.0 (1.2%). The results also revealed that there was a statistically significant association (χ^2^ = 12.5, P = 0.014) between the presence of *Cryptosporidium* oocysts in raw-eaten vegetables and vegetable types, and carrot had 5-times likelihood/odds (OR = 5.0, 95%CI = 1.07–23.5) of being more infectious than the others when consumed raw. Likewise, the garden egg is ten times more likely to be contaminated than the other vegetable types (Cucumber and Tomato) (Table 1).

**Table 1:** Prevalence of *Cryptosporidium* oocysts in raw eaten vegetables in MMC and Jere LGAs.

Vegetables	Negative (%)	Positive (%)	OR (95% CI)	χ^2^	p-value
Cabbage	70/80 (87.5)	10.0/80 (12.5)	Ref	12.5	*0.004
Carrot	78/80 (97.5)	2.0/80 (2.5)	*5.0 (1.07–23.5)		
Cucumber	74/0 (92.5)	6.0/80 (7.5)	1.7 (0.56–4.8)		
Garden egg	7/80 (98.8)	1.0/80 (1.2)	*10.0 (1.25–79.9)		
Tomato	71/80 (88.7)	9.0/80 (11.3)	1.1 (0.43–2.9)		
Total	372 (93.0)	28 (7.0)	400		

*Statistically significant value (P < 0.05).

### Distribution of *Cryptosporidium* oocysts in different locations of vegetables markets

Out of the five (5) different locations visited, Tashan Bama Market had the highest number 8.0 (8.0%) of vegetables with *Cryptosporidium* oocysts, followed by Gomboru Market and Unimaid Commercials with 7.0 (7.0%) and 3.0 (6.0%) respectively*.* However, there was a strong association, which was not statistically significant (P = 0.98), between the presence of *Cryptosporidium* oocysts and vegetable Markets’ in MMC and Jere LGAs (Table 2). Although, the odd ratios were protective among all the vegetable markets, signifying little or no risk.

**Table 2:** Distribution of* Cryptosporidium* oocysts in different vegetable markets in MMC and Jere LGAs.

Sample location	Negative (%)		Positive (%)	OR (95% CI)	χ^2^		P-value
202-vegetable-vendor	46.0/50 (92.0)		4.0/50 (8.0)	Ref	12.5		*0.98
Gomboru market	93.0/100 (93.0)		7.0/100 (7.0)	1.14 (0.32–4.1)			
Monday market	94.0/100 (94.0)		6.0/100 (6.0)	1.33 (0.36–4.9)			
Tashan Bama	92.0/100 (92.0)		8.0/100 (8.0)	1.00 (0.29–3.5)			
Unimaid commercials	47.0/50 (94.0)		3.0/50 (6.0)	1.33 (0.28–6.3)			
Total	372 (93.0)		28 (7.0)	400			

*Non-significant P-value (P > 0.05).

### Detection of *Cryptosporidium* oocysts from different vegetable sources in the study area

The vegetables were mainly sourced from the irrigation areas of MMC and Jere LGAs, which include Alau, Gorongo, Jetete, Kilari_Abdallahi, and Zabarmari*.* Among the sources of the vegetables sampled, Alau had the highest number of *Cryptosporidium* oocysts at 15.0 (12.5%), followed by Kilari-Abdullahi and Zabarmari sources with 4.0 (10.0%) and 4.0 (5.0%) respectively. However, Jetete appeared to have the least number, 2.0 (2.5%) of *Cryptosporidium* oocysts. The results also revealed a statistically significant association (χ^2^ = 10.4, P = 0.034) between the presence of *Cryptosporidium* oocysts and the sources of vegetables and fruits. Similarly, raw-eaten vegetables from Gorongo and Jetete had the odds of 3.0 and 5.0, respectively, meaning they are three and five times more likelihood of being more contaminated with *Cryptosporidium* oocysts than those from the other sources (Zabarmari and Kilari-Abullahi sites) (Table 3).

**Table 3:** Detection of *Cryptosporidium oocysts* in different vegetable sources in MMC and Jere LGAs.

Source	Negative (%)	Positive (%)	OR (95%CI)	χ^2^	P-value
Alau-Site	105.0/120 (87.5)	15.0/120 (12.5)	Ref	10.4	*0.034
Gorango	77.0/80 (96.2)	3.0/80 (3.8)	*3.3 (0.93–11.9)		
Jetete	78.0/80 (97.5)	2.0/80 (2.5)	*5.0 (1.11–22.5)		
Kilari-Abdullahi	36.0/40 (90.0)	4.0/40 (10.0)	1.2 (0.39–3.99		
Zabarmari-Site	71.0/80 (88.7)	9.0/80 (11.3)	1.1 (0.46–2.6)		
Total	372 (93.0)	28.0 (7.0)	400		

*Statistically significant P-value (P < 0.05).

## Discussion

*Cryptosporidium* oocysts are one of the most neglected foodborne pathogens of public health importance aside from other coliforms established in the food-chain transmission cycle in low-medium-income countries such as Nigeria^28^. The overall prevalence rate of *Cryptosporidium* oocysts found in this study was 7.0%, a significant public health threat. This is because vegetables and fruits are common delicacies in this region due to their affordability and accessibility. Similarly, consuming raw vegetables and fruits is common among consumers around the study area without considering the hygienic standard of the Vegetables and fruits. This habit may increase the likelihood of infection and occasionally diarrhoea in immunocompromised individuals associated with *Cryptosporidium* oocysts. The prevalence rate reported in this study appeared inconsistent with the findings of^29^, who reported a relatively lower prevalence rate of 4.42% in vegetables in Abuja, North-Central Nigeria. The disparity may be due to the socioeconomic literacy level coupled with good personnel hygiene among Abuja vegetable vendors compared to Maiduguri, where the majority had low socioeconomic status and low literacy level in addition to poor hygiene standards. However, a higher prevalence rate (14.5%) was reported by^30^ in the vegetable market of the semi-urban slum of Peru (South America). The semi-urban slum, where the study was conducted in Peru, is of lower socioeconomic status than other Sub-Saharan African Countries such as Nigeria. Therefore, there is a likelihood of getting more vegetables contaminated with oocysts due to poor hygiene standards^31^. Furthermore, the variations in prevalence rates could be due to differences in environmental conditions, hygienic measures adopted in their management, and methods of handling or processing the vegetables in different study areas reported^20,32^.

The result also revealed a statistically significant (p < 0.05, = 12.5) association between the presence of *Cryptosporidium* oocysts and raw-eaten vegetable types. This also revealed that the vegetable type could be a risk factor (OR = 5.0, 95%CI:1.07–23.5; OR = 10.0, (95%CI:1.25–79.9) for contracting *Cryptosporidium* infections in the food chain involving carrot and Garden egg vegetables with about 5 and 10 times likelihood than the other vegetable types, respectively. This suggests a risk of exposure for those who consume raw or improperly cooked vegetables of all types. This finding is in agreement with the reports of^33,34^, who also linked Cryptosporidiosis with vegetable type and habits of eating raw-eaten vegetables among the population at risk. Similarly, the contact of the vegetables with soil may also play a significant role in the contamination of the vegetables with *Cryptosporidium* oocysts as the vegetables were kept in a dirty environment and in contact with the soil. Likewise, fertilization of horticultural crops, including vegetables such as cabbage, tomatoes, carrots, etc., with manure from camel, cattle, and sheep, which may contain viable oocysts of *Cryptosporidium*, represents a significant risk to the contamination of vegetables as reported by^35^. The high level of contamination of *Cryptosporidium* in leafy vegetables than in root vegetables may result in continuous exposure to soil contaminants than the other vegetable types. This is also concord with the reports of other previous studies^9,34^. However, a similar study reported a contrary view of more contamination in the roots than in leafy vegetables ^33^. The reason for the higher level of contamination in cabbage may be attributed to the overlapping nature of the leaves, thereby creating a conducive microclimatic environment for the survival of the oocysts of the parasites compared to the other vegetable types in this study.

Regarding the sources of vegetables, *Cryptosporidium* oocysts were identified in raw vegetables from all the five markets sampled in this study. The fact that all the markets recorded positive samples suggests similar farming practices and common sources of vegetables sold for human consumption in Maiduguri Metropolis. On the other hand, the discrepancy in the occurrence of *Cryptosporidium* oocysts on vegetables from different markets reflects differences in hygienic practices during transportation and handling by the vendors in the food chain. Therefore, vegetables from the crowded markets vis-a-vis larger markets like Monday Market and Gamboru Vegetable Market had higher levels of contamination with *Cryptosporidium* oocysts than other locations. This may be attributed to poor water sources used for vegetable washing and other unhygienic practices among vegetable vendors. The *Cryptosporidium* oocysts identified in the vegetables from the study could have originated from the water source or manure used in the vegetable farms.

Furthermore, the major sources of vegetables in Maiduguri and its environs are Alau, Gorongo, Jetete, Kilari-Abdullahi*,* and Zabarmari sites. Most of the vegetable farmers in Maiduguri and environ utilize animal droppings, especially camel dung, poultry litter, and ruminants’ dung, as an organic manure for the cultivation of vegetables, which could be another source of oocysts contaminant and risk of exposure to the final consumers, if raw vegetables are consumed. This is similar to the report of^36^. Most of the vegetables in the study area came from the riverine areas surrounding the metropolis, such as Alau Dam, Dusuman River, Zabarmari Lake, etc. Therefore, exposure to *Cryptosporidium* oocysts is likely through the consumption of raw-eaten vegetables from these sources if not properly washed before being eaten. Moreover, the study revealed a significant risk of exposure to be 3 and 5 times more likely from Gorongo and Jetete, respectively, of being more contaminated with *Cryptosporidium* oocysts than the other sites. This may be attributed to their hygienic level, poor economic status, and educational background of those populations. This is in agreement with the report of^37^, who also linked these risk factors to *Cryptosporidium* infection in poor communities in China.

Among the sources of the vegetables sampled, Alau had the highest number of positive *Cryptosporidium* oocysts, and Jetete appeared to have had the least number of oocysts. There was also a statistically significant (P = 0.034) association between the presence of *Cryptosporidium* oocysts and the source of vegetables. Meanwhile, as a result, people buying vegetables from the Alau site have a higher risk of getting infected with *Cryptosporidium* oocysts compared to the other sources used in this study.

Alau is the major source of vegetables in Maiduguri Metropolis. Consequently, most vendors buy the vegetables from the Alau site with the possibly high number of oocysts because of socio-economic activities around the water source and indiscriminate waste disposal, which could also serve as a source of *Cryptosporidium* oocysts to the vegetables. Moreover, the habit of open defecation among certain groups that live at the fringes of the city may be washed off into surface water and contaminate shallow wells, boreholes, and rivers used for vegetable irrigation in Maiduguri with the possible exposure to *Cryptosporidium* oocysts. This scenario creates an avenue for potential contamination of vegetables from the source such as Jetete, which had five times (OR = 5.0, 95%CI: 1.1–22.5) more likelihood of contamination than the other sources and exposes the public to the risk of infection with *Cryptosporidium* oocysts. Previous surveys conducted by^38^ have shown that *Cryptosporidium* oocysts are found in all types of water sources, and their presence is more common in surface than underground waters, as documented by^39^. More so, the same study reported a statistically significant association between the source of vegetables and the presence of *Cryptosporidium* oocysts.

### Limitations of the study

This study had some limitations, such as the lack of a steady electricity supply in Maiduguri, which affected our research work during the microscopic identification of the oocyst. The lack of funds and support from the University was also a limitation to characterize further the *Cryptosporidium* oocysts isolated from the raw-eaten vegetables in Maiduguri. This study did not ascertain the hygienic practices of farmers and vendors, a further recommendation.

## Conclusion

The study concluded that the raw-eaten vegetables sampled in Maiduguri Metropolis were contaminated with *Cryptosporidium* oocysts, a neglected public health threat. The study also revealed that raw-eaten vegetables are risky to public health and should be cleaned and washed before consumption and ensure they are from clean sources.

The study recommends public enlightenment to ensure hygienic measures, such as the adoption of the HACCP principles in the food chain regarding raw-eaten vegetables. It also recommends further molecular detection of *Cryptosporidium* oocysts from raw-eaten vegetables in Maiduguri (Supplementary File-10.1038/s41598-023-49451-0.).

### Supplementary Information

Supplementary Information.

## Data Availability

This was uploaded as supplementary materials.
